# Groundwater Depth Drives Carbon Pools and Population Dynamics in Deep‐Rooted Desert Plants of a Hyper‐Arid Ecosystem

**DOI:** 10.1002/ece3.72395

**Published:** 2025-10-30

**Authors:** Caibian Huang, Zhiqiang Ma, Bo Zhang, Fanjiang Zeng, Shaomin Zhang

**Affiliations:** ^1^ Xinjiang Key Laboratory of Desert Plant Roots Ecology and Vegetation Restoration, Xinjiang Institute of Ecology and Geography Chinese Academy of Sciences Urumqi China; ^2^ State Key Laboratory of Ecological Safety and Sustainable Development in Arid Lands Urumqi China; ^3^ Cele National Station of Observation and Research for Desert Grassland Ecosystem in Xinjiang China; ^4^ Institute of Agricultural Resources and Environment Xinjiang Academy of Agricultural Sciences Urumqi China

**Keywords:** *Alhagi sparsifolia*, arid ecosystem, groundwater depth, non‐structural carbohydrates, population dynamics

## Abstract

Groundwater plays a crucial role in supporting vegetation in hyper‐arid regions, yet its regulatory effects on deep‐rooted plants remain poorly understood. This study examined *Alhagi sparsifolia* Shap., a groundwater‐dependent desert shrub at the southern Tarim Basin, over 4 years (2021–2024). We investigated how groundwater depth (2.5, 4.5, 11.0 m) influences population dynamics, biomass, and non‐structural carbohydrates (NSCs). Our findings reveal that groundwater depth critically regulates carbon allocation and plant performance. NSC content exhibited nonlinear responses, with soluble sugars and starch reduced at 4.5 m depth, but elevated at 11.0 m. While total carbon content remained stable, its seasonal and interannual fluctuations were significant. Population density decreased, while individual biomass increased with groundwater depth, indicating a density–growth trade‐off. At deeper depths, individual carbon pools expanded, but overall population biomass and carbon pools declined, suggesting a recruitment threshold. Random forest and PLS‐PM analyses identified groundwater depth and soil moisture as key drivers of population demographic and metabolic traits. These results underscore the pivotal role of carbon metabolism and recruitment in shaping the ecological strategies of desert plants under water scarcity.

## Graphical abstract

Groundwater depth shaped A. sparsifolia density and individual biomass. Carbon pools aided population persistence but couldn't offset deep groundwater's negative impact. Groundwater limits exceeded climate effects on desert vegetation persistence.

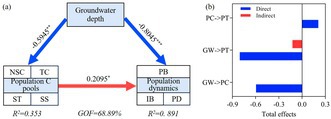

## 1 Introduction

Groundwater is the largest reservoir of liquid freshwater on Earth, supporting approximately 37% of global terrestrial vegetation, particularly in arid and semi‐arid ecosystems (Barbeta and Peñuelas 2017). However, accelerating anthropogenic pressures have led to widespread groundwater depletion, especially in dryland regions (de Graaf et al. 2019; Shakya et al. 2019; Jia et al. 2020). In hyper‐arid environments, land development and intensive groundwater extraction have significantly deepened groundwater levels, contributing to increased soil salinization (Wang et al. 2023). These hydrological changes profoundly disrupt ecosystem processes, altering biogeochemical cycles and vegetation composition, structure, and function (Wang et al. 2021; Xie and Li 2022; Dong et al. 2023), ultimately threatening ecosystem resilience and regional sustainability. Despite these challenges, species‐specific ecohydrological responses, driven by ecological niche differentiation, may enable adaptive strategies and functional adjustments under groundwater stress (Araya et al. 2011; Silvertown et al. 2015). Understanding how groundwater dynamics influence vegetation processes is critical for predicting ecosystem responses in increasingly arid environments.

With ongoing climate change and intensifying anthropogenic disturbances, regional droughts and groundwater declines are expected to persist, driving progressive shifts in vegetation morphology, abundance, and community composition (Froend and Sommer 2010; Sommer and Froend 2011; Mu et al. 2024). Morphological adaptations typically include reductions in plant height, canopy area, and basal diameter (Wu et al. 2024), while some xerophytic species exhibit leaf reduction into membranous scales, with photosynthesis shifting to photosynthetic stems (Liu et al. 2016). Abundance and diversity typically decrease as a result of increased mortality and population decline, with shifts favoring drought‐tolerant species that often rely on shallow groundwater sources (Sommer and Froend 2011). Species‐specific thresholds for groundwater availability, beyond which mortality and community degradation occur, drive these responses (Horton et al. 2001; Feng et al. 2024). For example, *Haloxylon ammodendron* was unable to survive with groundwater depths exceeding 15 m and was replaced by the more drought‐tolerant *H. persicum* (Mu et al. 2024). While some species can mitigate drought stress through eco‐physiological adjustments, the benefits of these strategies are often constrained by ecological costs (Barbeta et al. 2013; Xu et al. 2023; McDowell et al. 2008).

Declining groundwater levels could profoundly alter plant growth in terrestrial ecosystems, affecting both the accumulation and allocation of aboveground and belowground biomass (Wang, Zeng, et al. 2018; Mao et al. 2022; He et al. 2025), and ultimately influencing the carbon sequestration potential of vegetation (Mäkiranta et al. 2018). Plant biomass responses to lowered groundwater levels, however, vary widely among species—ranging from increases to declines—while root‐to‐shoot ratios generally tend to rise (Wang, Yu, et al. 2018; Wu et al. 2024; He et al. 2025). Groundwater‐driven hydrological drought also severely impaired fundamental plant physiological processes, reducing both water content and carbon acquisition capacity (Rossatto et al. 2012; Antunes et al. 2018). Non‐structural carbohydrates (NSCs)—primarily soluble sugars and starch—act as dynamic regulators of the plant source–sink balance (Martínez‐Vilalta et al. 2016; Hartmann et al. 2020), serving as crucial buffers that support survival under environmental stresses such as drought and low temperatures (O'Brien et al. 2014; Blumstein et al. 2023). NSCs serve dual roles in maintaining carbon supply during periods of limited photosynthesis and osmotic regulation through soluble sugars (McDowell et al. 2008; Dietze et al. 2014). Evidence from arid ecosystems showed that groundwater declines could lead to a significant increase in soluble sugar concentrations and total NSC levels in leaves or assimilating shoots (Zhao, Chang, et al. 2003; Feng et al. 2022). Although such increases may come at the cost of reduced growth rates, they can enhance plant adaptive capacity under stress (O'Brien et al. 2014). In addition, seasonal fluctuations in NSC pools support critical growth processes, such as bud break and leaf growth in early spring (Schädel et al. 2009; Zhao et al. 2023). Consequently, NSC storage capacity is increasingly recognized as influencing the growth–survival trade‐offs under multiple biotic and abiotic stresses, with emerging evidence suggesting its predictive value for plant survival or mortality under carbon stress (Plotkin et al. 2021; Zhang et al. 2024).

*Alhagi sparsifolia* Shap., a perennial clonal legume endemic to northwestern China, dominates desert vegetation in the southern Tarim Basin. This species reproduces primarily via clonal propagation, forming large patches of up to 6.1 hm^2^ (Vonlanthen et al. 2010). *A. sparsifolia* has adapted to prolonged arid and high‐temperature conditions, with its photosynthesis mainly occurring in current‐year green stems due to severe leaf reduction. It relies on an extensive deep‐root system to access groundwater (Arndt et al. 2004) and demonstrates high regenerative capacity via adventitious sprouting, contributing to sand stabilization and providing forage and medicinal resources. In recent decades, *A. sparsifolia* populations along the southern margin of the Tarim Basin have shown varying degrees of degradation; yet whether groundwater table decline is the primary driver of this trend remains unclear. Controlled experiments have demonstrated that lowered groundwater tables reduce aboveground and whole‐plant biomass accumulation in seedlings (Li et al. 2015), while promoting greater biomass allocation to belowground structures (Zeng et al. 2013; Li et al. 2015). In contrast, field investigations suggest that mature plants exhibit a different response: individual biomass increases under moderate groundwater depths (Zhang et al. 2018), with no significant change in population‐level aboveground biomass, yet a clear increase in root biomass with declining groundwater levels (Gui et al. 2013). Furthermore, limited evidence indicates that both seedlings and adult plants exhibit enhanced photosynthetic capacity under lower groundwater tables (Zhang et al. 2011; Wang, Yu, et al. 2018). Despite these findings, the role of NSC reserves in mediating the physiological and growth responses of *A. sparsifolia* to groundwater table fluctuations remains poorly understood. Elucidating this link is critical for advancing our understanding of carbon allocation strategies in long‐lived clonal plants under arid conditions, and for predicting the persistence and functional stability of dominant desert species under scenarios of groundwater depletion driven by climate change and intensified water extraction.

This study aims to investigate the growth adaptations of *A. sparsifolia* populations under contrasting groundwater depths in the southern Tarim Basin over 4 years. The objectives are to (1) assess changes in population stability and carbon pool dynamics in response to groundwater decline; (2) analyze the seasonal variation of NSCs and their relationship with population biomass; and (3) explore the connection between carbon storage capacity and population stability, revealing the carbon metabolism and growth adaptation strategies of *A. sparsifolia* under groundwater depletion. This research provides empirical insights into predicting the stability and functionality of desert vegetation in the face of climate change and intensified human disturbances.

## 2 Materials and Methods

### 2.1 Study Area Description

The experimental site is located at the oasis‐desert ecotone along the southern edge of the Tarim Basin (37 00′ N, 80 43.45′E), with an average elevation of 1366 m. The region experiences a typical continental desert climate, characterized by a mean annual precipitation of 42.6 mm (over 70% concentrated in summer), an annual potential evaporation of 2751.6 mm, and a mean annual temperature of 15.9°C. In summer (June–August), mean daily air temperatures usually range between 25.0°C and 27.0°C, while extreme maximum temperatures can reach 41.9°C. Diurnal temperature variation is pronounced. Vegetation is sparse, dominated by groundwater‐dependent perennials. *A. sparsifolia* is the dominant species, accompanied by *Karelinia caspica*, *Phragmites communis* , and *Tamarix ramosissima* . Soils are classified as Arenosols (FAO/ISRIC/ISSS, 1998), composed predominantly of 95%–98% silt and very fine sand (Mao et al. 2015), and characterized by mild salinization and low nutrient content (Zhang et al. 2018).

Groundwater is primarily recharged by the Cele River, a glacier‐fed river originating from the Kunlun Mountains. The long‐term average annual runoff is 1.43 × 10^8^ m^3^, increasing to 1.63 × 10^8^ m^3^ between 2019 and 2023. Groundwater depth follows a general south‐to‐north gradient, being shallower in the north (Qian et al. 2018). Long‐term monitoring at 25 wells indicated groundwater depths ranging from 2.0 to 15.0 m, with a mean annual variation of 0.06 m over the past 4 years, suggesting relative stability.

**FIGURE 1:**
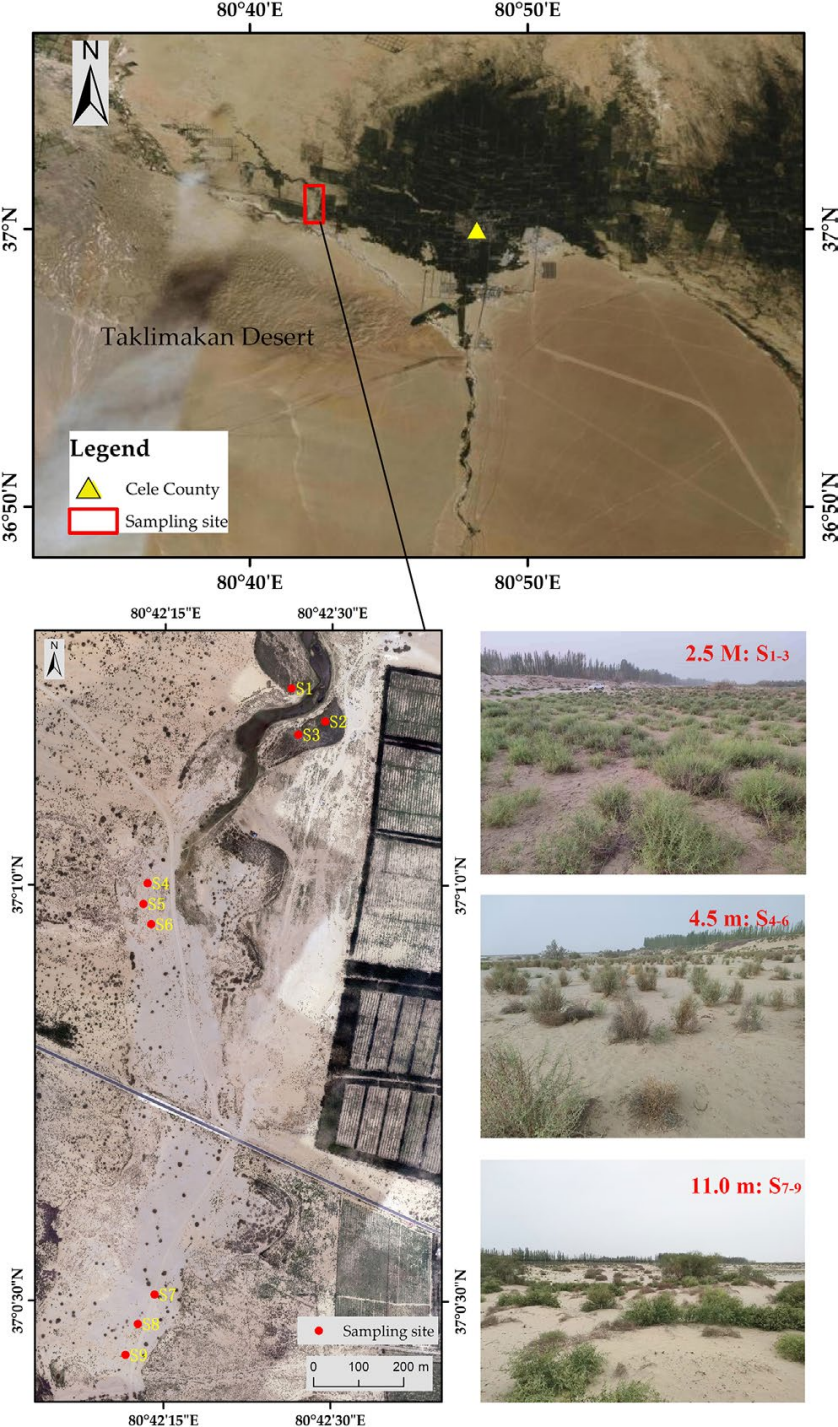
Sampling sites and landscape of the groundwater depths of 2.5, 4.5, and 11.0 m, respectively.

Field surveys in arid regions have shown that the optimal groundwater table depth for the growth of *A. sparsifolia* is approximately 2–4 m (Zhang et al. 2004). Considering local groundwater conditions and the potential disturbance to plant communities, three representative groundwater table depths—2.5, 4.5, and 11.0 m—were selected in the study area as observation gradients, following the approach of Gui et al. (2013). Previous root profile excavations indicated that, at each of these groundwater depths, the taproots of *A. sparsifolia* were directly connected to the groundwater (Gui et al. 2013). Based on this framework, in mid‐July 2021, we established nine typical study plots across the three groundwater table depths, each exhibiting marked differences in vegetation density and minimal anthropogenic disturbance (> 20 years of protection). Sites S_1_–S_3_ (2.5 m groundwater depth; 37°01′12" N–80°42′29" E), S_4_–S_6_ (4.5 m; 37°01′59" N–80°42′13" E), and S_7_–S_9_ (11.0 m; 37°00′28" N–80°42′13" E) were selected to represent high, medium, and low groundwater availability, respectively (Figure 1). Detailed descriptions of vegetation composition, community structure, and soil properties for each groundwater depth can be found in Gui et al. (2013) and Zhang et al. (2018). Over the monitoring period from 2021 to 2024, the groundwater levels at depths of 2.5, 4.5, and 11.0 m demonstrated average annual variations of 0.10, 0.07, and 0.12 m, respectively.

Meteorological data (monthly precipitation and temperature) from 2021 to 2024 were obtained from the Cele National Station (Figure S1). Annual rainfall during this period ranged from 17.7 to 38.9 mm (mean: 28.68 mm), with approximately 70% occurring between June and August. Winter precipitation in the region occurs primarily in the form of snowfall. Mean annual temperatures varied from 12.2°C in 2021 to 13.1°C in 2022, with extremes of 40.5°C (July 2023) and −20.1°C (January 2021).

### 2.2 Field Surveys and Sample Collection

#### 2.2.1 Plot Establishment and Vegetation Survey

Field surveys were conducted in July 2021 across a groundwater depth gradient. The terrain was flat, minimizing topographic influences. At each site (about 2.0 hm^2^), three sampling points were randomly located at intervals of at least 50 m, yielding nine points (S_1_–S_9_). At each point, two adjacent 10 m × 10 m fixed plots were established, separated by 10 m to serve as biological replicates. Living *A. sparsifolia* individuals within plot boundaries were recorded, and plant density (plants/hm^2^) along with growth parameters was measured. To capture interannual dynamics, surveys were repeated each July from 2022 to 2024.

#### 2.2.2 Aboveground Biomass Sampling and Measurement

To minimize disturbance during the windy season (March–September), a dispersed sampling strategy was used to maintain plot integrity. During peak biomass (late August each year), standard individuals were selected based on plant height and canopy area (major × minor axes). Three representative individuals per plot were harvested from the periphery (≥ 2 m from plot boundaries). Aboveground parts were weighed for fresh mass, then oven‐dried at 105°C for 30 min, followed by drying at 75°C to a constant weight to determine the dry mass. Individual biomass was calculated as dry aboveground biomass per plant (g/plant). Aboveground water content (%) was expressed as the proportion of water lost during drying, calculated as the difference between fresh mass and dry mass divided by fresh mass, multiplied by 100. Population biomass (kg/hm^2^) was estimated by multiplying mean individual biomass by plant density.

#### 2.2.3 Carbon Content and Carbon Pool Assessment

*A. sparsifolia* follows a seasonal growth pattern, with bud sprouting in early April, branching in early May, rapid biomass accumulation from June to August, and complete senescence by January of the following year, which is typically the coldest month. To capture seasonal carbon dynamics, healthy green branches (100 g per plot) were collected at maturity (late August) each year from 2021 to 2024. Senesced branches were sampled in January, April, and May. Samples were dried, ground, and lyophilized. Soluble sugar and starch contents (%) were determined using the anthrone colorimetric method (Yemm and Willis 1954; Zhou et al. 2021), while total carbon (TC) content was measured using the Walkley‐Black dichromate oxidation method (Nelson et al. 1982). Non‐structural carbohydrate (NSC) content (%) was calculated as the sum of soluble sugars and starch. Individual NSC storage (g/plant) was calculated by multiplying NSC content by biomass, and population‐level carbon pools (kg/hm^2^) were derived by scaling from individuals to population level.

#### 2.2.4 Soil Moisture Monitoring

Considering that *A. sparsifolia* horizontal spacers between cloned ramets typically do not exceed 1.0 m depth, soil moisture was assessed within the 0–120 cm profile. In late August from 2021 to 2023, soil samples were collected at 20 cm increments within each plot. Gravimetric soil water content (%) was measured after oven‐drying at 105°C. Results indicated significantly lower soil water content at the 4.5 and 11.0 m groundwater sites compared to the 2.5 m site (Figure S2).

### 2.3 Data Analysis

All statistical analyses were performed using SPSS 21.0 (SPSS Inc., Chicago, IL, USA). Linear mixed‐effects models were used to assess the effects of groundwater depth (fixed factor), month, year, and their interactions on branch carbon components (NSC, starch, soluble sugars, TC) and ratio parameters (soluble sugar/starch (SS/ST), NSC/TC). Significant main effects (*p <* 0.05) were further analyzed using Duncan's multiple range tests.

One‐way ANOVA was used to evaluate the independent effects of groundwater depth and year on aboveground carbon pools, biomass, and density, followed by Duncan's post hoc tests. Pearson correlation analysis, performed in Origin 2021 (OriginLab Corp., Northampton, MA, USA), was used to quantify relationships between carbon components, ratio parameters, population density, and biomass.

To explore the environmental determinants of population biomass and carbon pools (NSC, TC), random forest regression (RandomForest package) was applied, with the percentage increase in mean squared error (%IncMSE) used to evaluate variable importance. Partial least squares path modeling (PLS‐PM) (plspm v4.0.1, R package) was further employed to assess the regulatory effects of groundwater depth and current‐year aboveground carbon pools on subsequent‐year population density and biomass. All figures were generated using GraphPad Prism 9.0 (GraphPad Software Inc., San Diego, CA, USA).

## 3 Results

### 3.1 Total Carbon and NSC Contents in Current‐Year Branches

**FIGURE 2:**
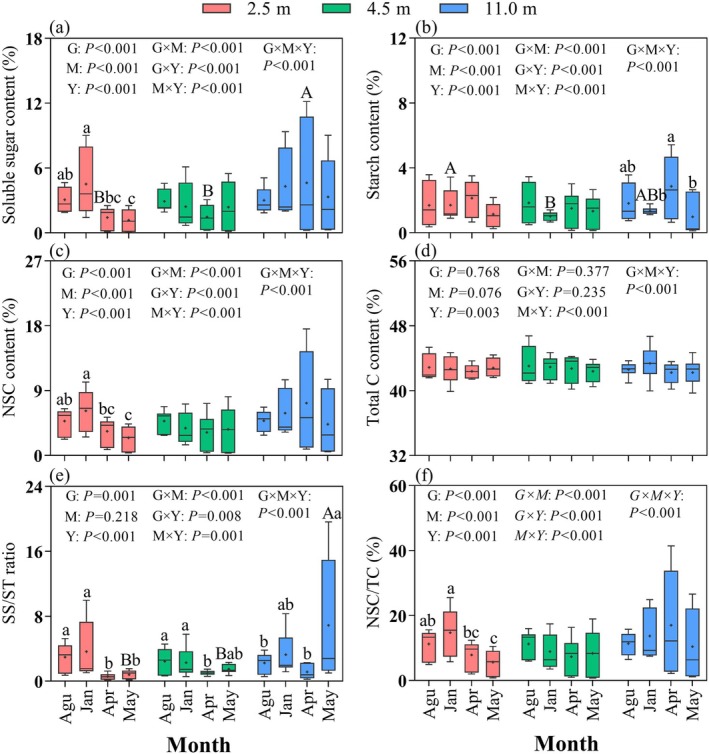
Influence of groundwater depth and sampling month on the contents of total carbon (C), non‐structural carbohydrate (NSC), and its components in current‐year shoots of *Alhagi sparsifolia* Shap. from 2021 to 2023. Data are means ± SE, *n* = 9. G × M × Y, interaction among groundwater depth, month and year; G × M, interaction between groundwater depth and month; G × Y, interaction between groundwater depth and year; G, groundwater depth; M × Y, interaction between month and year; M, month; SS, soluble sugars; ST, starch; TC, total carbon; Y, year. The black cross symbol within the box represents the mean value. Different small letters represent significant differences among months at *p* < 0.05. Different capital letters represent significant differences among groundwater depths at *p* < 0.05.

Groundwater depth exerted a nonlinear effect on non‐structural carbohydrate (NSC) content and its components in the current‐year branches of *A. sparsifolia* (Table 1, Figure 2). At a moderate groundwater depth of 4.5 m, soluble sugar and starch contents were significantly reduced (*p* < 0.001). In contrast, at 11.0 m, soluble sugar, NSC content, and the ratios of soluble sugar to starch (SS/ST) and NSC to total carbon (NSC/TC) increased significantly (*p* < 0.001). Total carbon content remained relatively stable across groundwater depths (Table 1). The interaction between month and year contributed to greater variability in NSC dynamics (Figure 2a–c).

**TABLE 1:** Variations in the contents of total carbon (C), non‐structural carbohydrate (NSC), and its components in current‐year shoots of *Alhagi sparsifolia* on the 3‐year (i.e., 2021–2023) average at different groundwater depths, months, and years (mean ± SE).

Factors	Treatment	SS (%)	ST (%)	NSC (%)	TC (%)	SS/ST (ratio)	NSC/TC (%)
Groundwater depth	2.5 m	2.54 ± 2.16b	1.67 ± 1.06a	4.18 ± 2.47b	42.69 ± 1.30a	2.00 ± 2.33b	9.86 ± 6.03b
	4.5 m	2.30 ± 1.69c	1.43 ± 0.95b	3.83 ± 2.30b	42.77 ± 1.63a	1.82 ± 1.30b	8.95 ± 5.44b
	11.0 m	3.81 ± 3.37a	1.75 ± 1.38a	5.57 ± 4.21a	42.61 ± 1.48a	3.39 ± 4.44a	13.11 ± 10.02a
Month	Aug.	3.00 ± 1.05b	1.78 ± 1.17b	4.78 ± 1.54b	42.84 ± 1.60ab	2.56 ± 1.49b	11.26 ± 3.83b
	Jan.	3.75 ± 2.84a	1.36 ± 0.63c	5.28 ± 2.81a	42.99 ± 1.67a	3.07 ± 2.65a	12.44 ± 6.95a
	Apr.	2.50 ± 3.30c	2.17 ± 1.44a	4.60 ± 4.45b	42.45 ± 1.25b	0.92 ± 0.61c	10.72 ± 10.42b
	May.	2.29 ± 2.43c	1.16 ± 0.95d	3.46 ± 3.09c	42.48 ± 1.29b	3.07 ± 5.03a	8.13 ± 7.45c
Year	2021	2.13 ± 0.68b	2.17 ± 1.12a	4.44 ± 1.43b	42.94 ± 0.89a	2.32 ± 4.46b	10.38 ± 3.44b
	2022	5.49 ± 2.80a	2.02 ± 1.06b	7.45 ± 3.21a	42.26 ± 1.18b	3.26 ± 2.23a	17.68 ± 7.67a
	2023	1.03 ± 0.94c	0.66 ± 0.41c	1.69 ± 1.20c	42.88 ± 2.02a	1.63 ± 1.44c	12.25 ± 9.29c

*Note:* Different small letters mean significant differences among groundwater depths, months, and years at *p* < 0.05.

Abbreviations: SS, soluble sugars; ST, starch; TC, total carbon.

Soluble sugar content varied significantly with groundwater depth only in April, where the 11.0 m depth exhibited higher values compared to shallower depths (*p* < 0.05, Figure 1a). Starch content responded to groundwater depth only in January, being significantly lower at 4.5 m than at 2.5 m (*p* < 0.01, Figure 1b). The SS/ST ratio showed significant differences in May, with values at 11.0 m significantly exceeding those at shallower depths (*p* < 0.001, Figure 1e). No significant effects of groundwater depth were observed for NSC concentration, total carbon content, or the NSC/TC ratio (Figure 2c,d,f).

Seasonal variation had a strong influence on carbon metabolic parameters (*p* < 0.001). Soluble sugar, NSC, total carbon content, and the NSC/TC ratio peaked in January (3.75%, 5.28%, 42.99%, and 12.44%, respectively), decreased sharply during sprouting in April, and reached their lowest values in May (Table 1). In contrast, starch content peaked in April (2.17%) and declined by 46.54% by May. The SS/ST ratio remained similar between January and May (3.07), but was significantly higher than in other months (*p* < 0.05, Table 2).

**TABLE 2:** Variations in the Individual and population C pools on the 3‐year (i.e., 2021–2023) average at different groundwater depths and years (mean ± SE).

Factors	Treatment	Individual C pools (g/plant)				Population C pools (kg/hm^2^)			
		Sugar	Starch	NSC	Total C	Sugar	Starch	NSC	Total C
Groundwater depth	2.5 m	3.86 ± 1.33b	2.27 ± 1.96b	6.16 ± 2.79b	54.30 ± 7.06b	73.08 ± 24.69a	43.06 ± 37.67a	116.67 ± 52.87a	1048.07 ± 206.49a
	4.5 m	4.73 ± 1.15b	3.18 ± 2.36b	7.91 ± 2.59b	72.44 ± 12.44b	26.42 ± 5.52b	18.49 ± 14.85a	44.91 ± 16.62b	412.26 ± 103.66b
	11.0 m	11.02 ± 6.41a	6.02 ± 3.10a	17.06 ± 7.19a	147.68 ± 33.85a	38.84 ± 26.16b	22.91 ± 15.31a	61.80 ± 32.97b	508.83 ± 188.43b
Year	2021	5.01 ± 1.47b	6.81 ± 2.19a	11.84 ± 3.53a	88.23 ± 31.39a	43.75 ± 25.15ab	56.29 ± 27.34a	100.52 ± 53.90a	720.00 ± 343.53a
	2022	8.18 ± 6.92a	5.26 ± 2.12b	13.45 ± 9.02a	100.58 ± 67.93a	50.69 ± 30.02a	29.99 ± 7.47b	80.74 ± 36.77a	602.82 ± 292.78a
	2023	7.17 ± 2.02b	3.59 ± 0.88c	10.77b±2.94a	97.41 ± 33.47a	44.90 ± 14.60b	20.05 ± 2.81b	65.02 ± 17.26b	615.96 ± 375.23a

*Note:* Different small letters mean significant differences among groundwater depths, months, or years at *p* < 0.05.

There was a significant interactive effect of month and groundwater depth on the carbon metabolism parameters of current‐year branches (*p* < 0.001, Figure 2). At 2.5 m, both soluble sugar content and the NSC/TC ratio showed significant monthly variation (*p* < 0.05, Figure 2a,c), whereas starch content exhibited significant monthly variation only at 11.0 m (*p* < 0.05, Figure 2b). The SS/ST ratio fluctuated monthly at both 2.5 and 4.5 m, being higher in January and August than in April and May (*p* < 0.05), and peaked in May at 11.0 m (Figure 2e).

**FIGURE 3:**
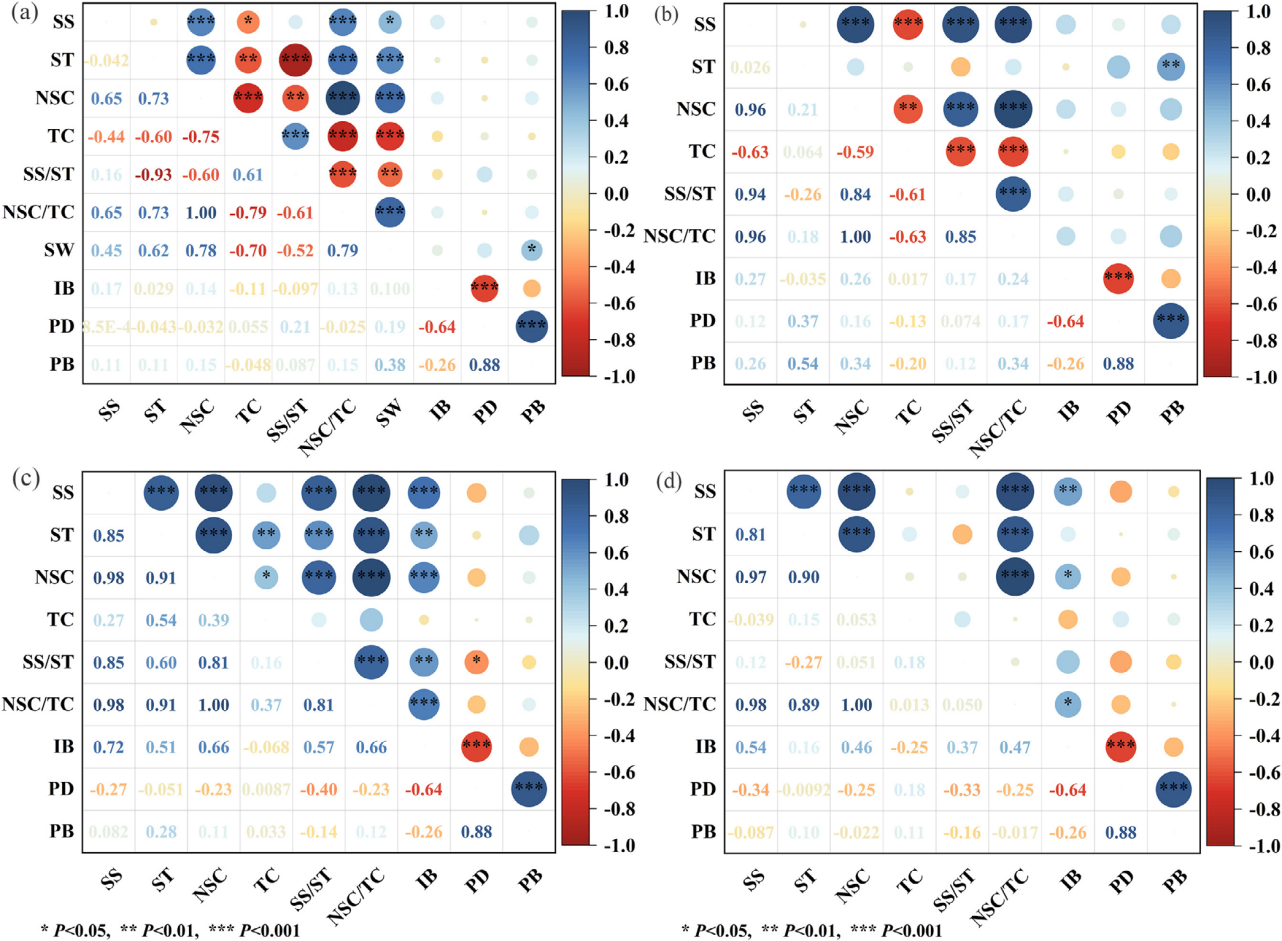
Pearson correlations among total carbon (TC), non‐structural carbohydrates (NSC) and their components, plant water content (SW), and key population parameters measured in August of the current year (a) and in January (b), April (c), and May (d) of the following year. SS, soluble sugars; ST, starch; IB, individual biomass; PB, population biomass; PD, population density.

Seasonally dependent decoupling of correlations among carbon components was observed (Figure 3). Soluble sugars, NSC, and the NSC/TC ratio were strongly positively correlated across all sampling months (*p* < 0.01). Total carbon content showed negative correlations with soluble sugars and NSC in August and January (*p* < 0.01, Figure 3a,b), but shifted to positive correlations with starch and NSC in April (*p* < 0.05, Figure 3c). No significant correlations were detected in May (*p* > 0.05, Figure 3d). Aboveground water content was positively correlated with soluble sugars, starch, NSC, and the NSC/TC ratio (*p* < 0.05, Figure 3a), but negatively correlated with total carbon and the SS/ST ratio (*p* < 0.01), suggesting that water stress influences carbon dynamics primarily through osmotic adjustment.

### 3.2 Individual and Population Biomass

**FIGURE 4:**
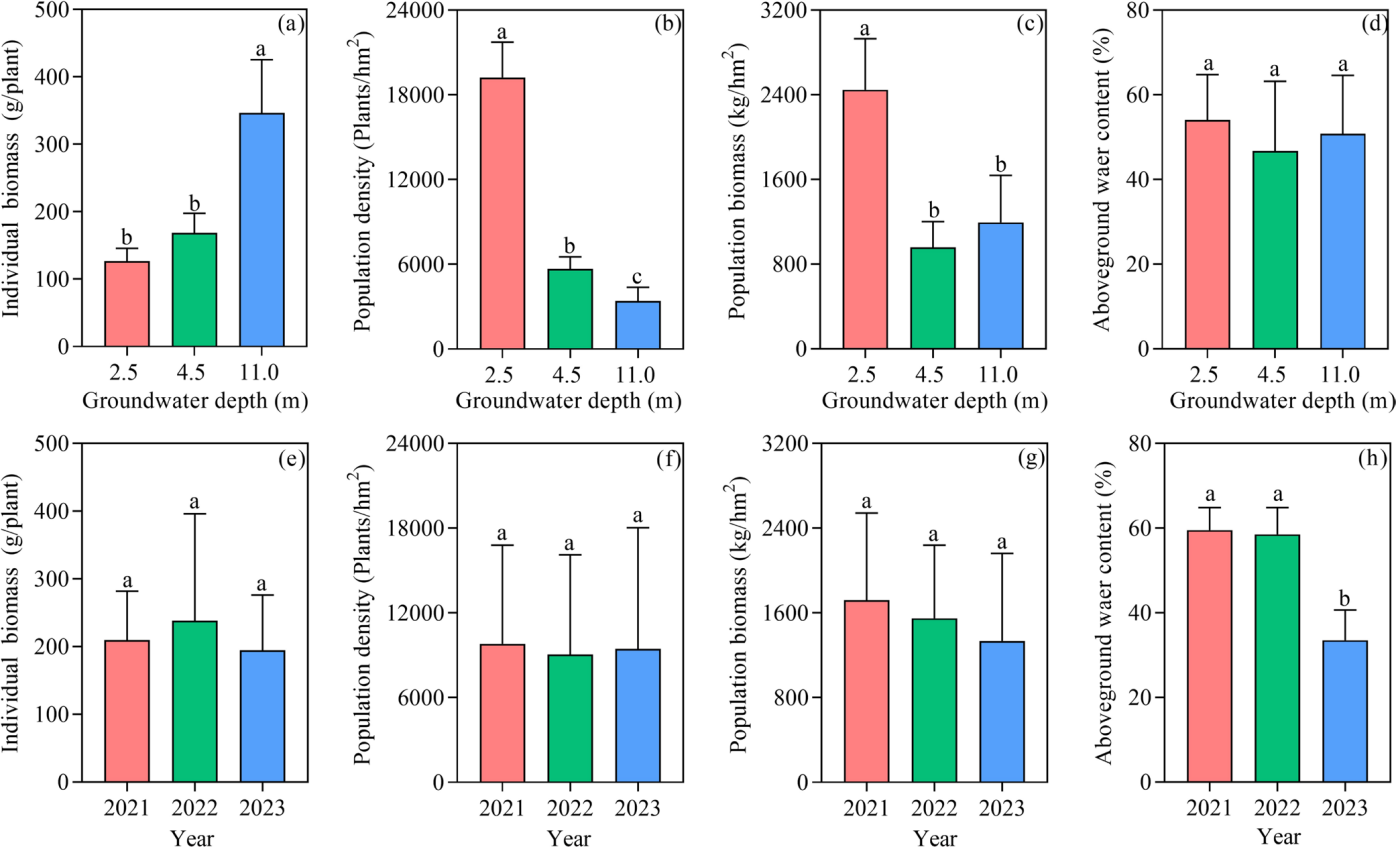
Variations in population density, biomass, and aboveground water content on the 3‐year (i.e., 2021‐2023) average at different groundwater depths and years (mean ± SE). Different small letters mean significant differences among treatments at *p* < 0.05.

Groundwater depth influenced the dynamics of population density and individual biomass of *A. sparsifolia* in a trade‐off manner (Figure 4). As groundwater depth increased, individual biomass significantly increased (*p* < 0.05, Figure 4a), whereas both population density and total population biomass decreased (*p* < 0.05, Figure 4a,b). No significant differences were observed between the 4.5 m and 11.0 m depths. The effects of groundwater depth on individual biomass and population density were consistent across years (Figure S3a,d).

Interannual variability in population biomass was notable. In 2021 and 2023, biomass followed the overall trend across groundwater depths, while in other years, biomass at 11.0 m exceeded that at 4.5 m (*p* < 0.05) but remained lower than at 2.5 m (Figure S3b). Interannual variations in mean individual biomass, population density, and total population biomass were generally not significant (Figure 4e–g).

The effect of groundwater depth on aboveground water content in *A. sparsifolia* was not significant overall (Figure 4d), but exhibited interannual fluctuations (*p* < 0.05, Figure 4h). In 2021–2022, water content did not differ among groundwater depths, but in 2023–2024, it was significantly lower at 4.5 m compared to other depths (*p* < 0.05), with no significant difference between 2.5 m and 11.0 m (Figure S3c). Overall, aboveground water content declined continuously from 2021 to 2023, with the sharpest decrease occurring in 2023, likely due to regional climatic factors such as temperature variations (Figure 4h).

Correlation analysis revealed a strong positive relationship between population biomass and population density (*p* < 0.001), but no significant correlation between population biomass and individual biomass (Figure 3). In contrast, individual biomass was strongly negatively correlated with population density (*p* < 0.001). Aboveground water content was positively correlated with population biomass (*p* < 0.05), but showed no significant relationship with individual biomass or population density (Figure 3a). Furthermore, the contents of carbon components in current‐year branches (soluble sugars, starch, NSC, and total carbon) were not significantly correlated with individual biomass, population biomass, or population density.

### 3.3 Population Total Carbon and NSC Pools

The individual carbon pools (soluble sugars, starch, NSC, and total carbon) in the aboveground biomass of *A. sparsifolia* increased significantly with declining groundwater depth, with the highest values consistently observed at 11.0 m (*p* < 0.05; Table 2, Figure S4a,c,e,g). A significant effect of year was detected for all individual carbon pools (*p* < 0.05). Soluble sugar and NSC pools peaked in 2022, while starch pools declined over the years (Table 2). There was a significant interaction between groundwater depth and year (Figure S4). Specifically, the NSC pool declined progressively at 2.5 m and 4.5 m depths (*p* < 0.05; Figure S4e), whereas it peaked sharply at 11.0 m in 2022 (*p* < 0.001). Total carbon pools showed no significant interannual variation at 2.5 m and 4.5 m depths but reached a maximum at 11.0 m in 2022 (*p* < 0.05, Figure S4g).

In contrast, groundwater depth significantly reduced population‐level carbon pools (soluble sugars, starch, NSC, and total carbon; *p* < 0.01), although no significant differences were found between the 4.5 m and 11.0 m depths (Table 2). Population carbon pool differences across groundwater depths showed strong interannual variability. In 2021 and 2023, differences followed the overall trend (Figure S4b,d,f,h). However, in 2022, carbon pools were highest at 2.5 m, though differences between 2.5 and 4.5 m were not statistically significant.

Interannual effects on population carbon pools were significant (*p* < 0.05). Soluble sugar pools peaked in 2022, while starch and NSC pools declined each year, and total carbon pools reached their minimum in 2022 (Table 2). For each groundwater depth, interannual variation in soluble sugar and starch pools followed the same trends as the overall means (Figure S4b,d), and NSC and total carbon pools at 2.5 and 4.5 m also conformed to the mean patterns (Figure S4f,h).

### 3.4 Control Parameters for Population Growth and Carbon Pools

**FIGURE 5:**
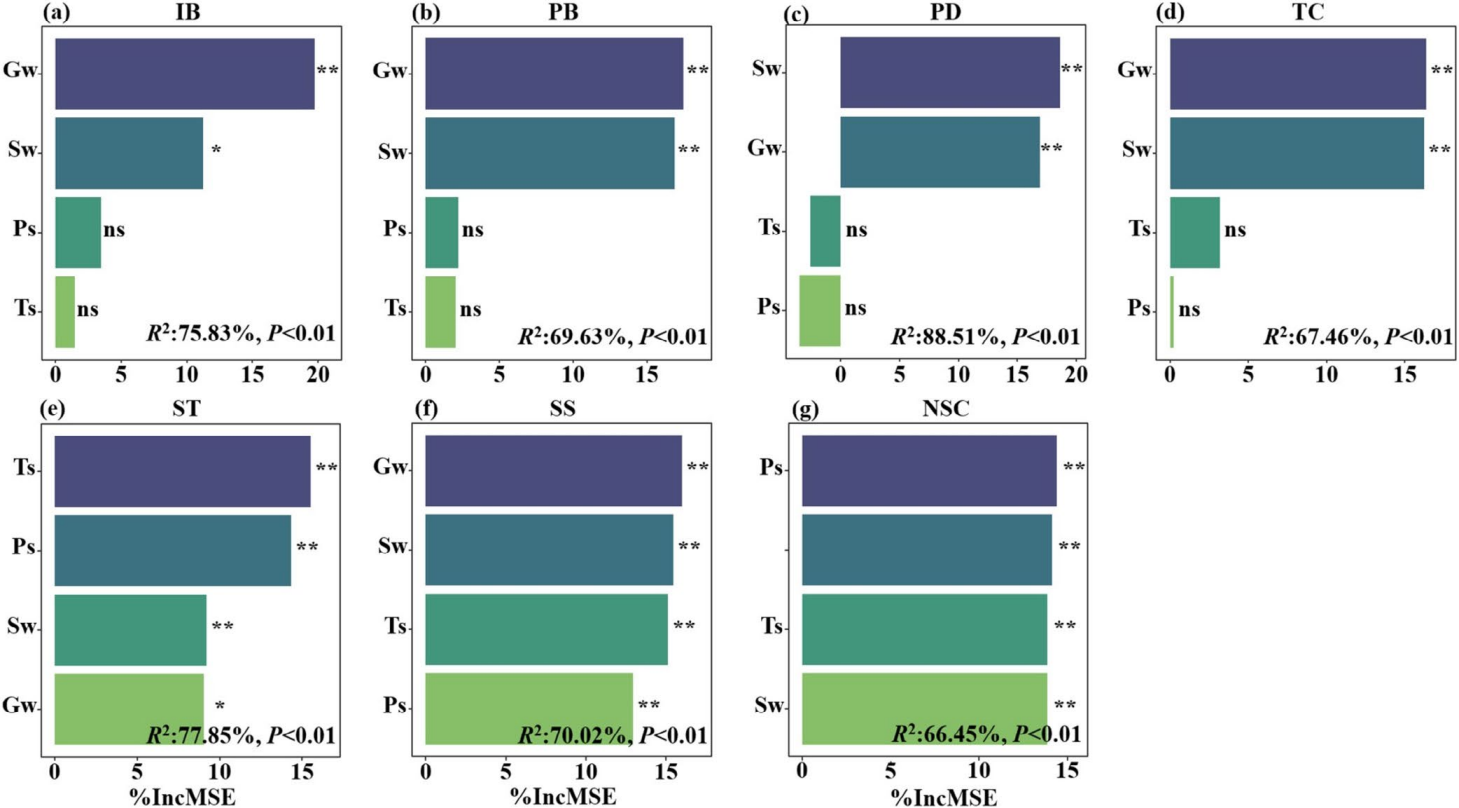
Random forest analysis to identify the importance ranking of environmental factors influencing population biomass, density, and C pools. The percent increase in mean squared errors (%IncMSE) represents the importance of main factors. *p < 0.05; **p < 0.01; ***p < 0.001. Gw, groundwater depth; IB, individual biomass; PB, population biomass; PD, population density; Ps, annual precipitation; SS, soluble sugars; ST, starch; Sw, average soil water content in 0–120 cm soil depth; TC, total carbon; Ts, average annual temperature.

Random forest models identified groundwater depth and soil moisture as the primary environmental drivers regulating the dynamics of *A. sparsifolia* population density, biomass, and carbon pools (Figure 5). These two variables accounted for the greatest proportion of variance in individual biomass (30.98%), population density (35.51%), population biomass (34.34%), total carbon pool (32.61%), and soluble sugar pool (31.43%, Figure 5a–d,f). Temperature and rainfall had secondary effects, primarily influencing starch (29.81%) and NSC pools (28.52%). Specifically, temperature regulated starch dynamics, while rainfall influenced NSC fluctuations, although neither factor significantly affected population density or biomass.

**FIGURE 6:**
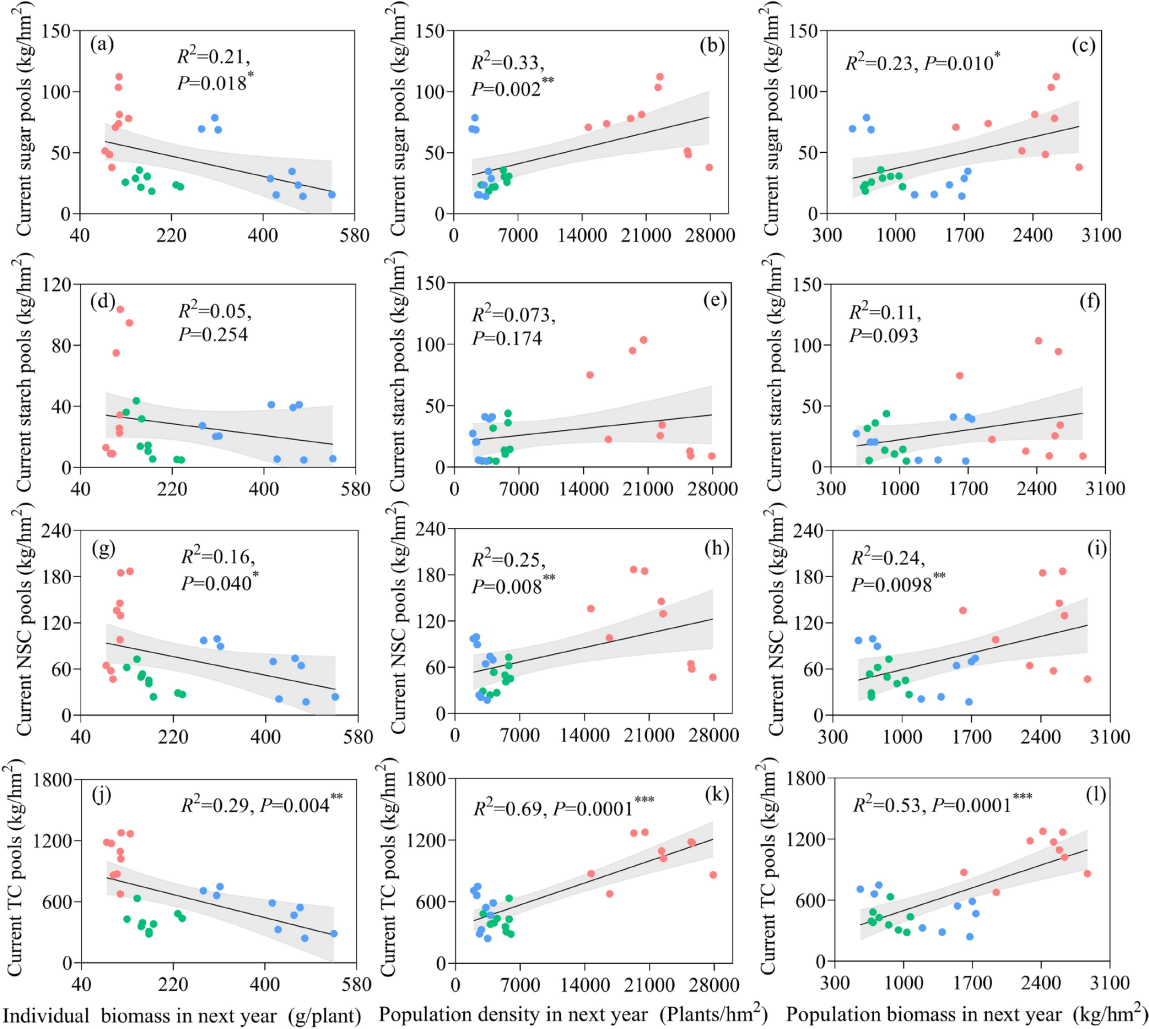
Relationships between current population C pools and next‐year population density and biomass. The solid lines represent trendlines. Red ball, at groundwater depth of 2.5 m; green ball, at groundwater depth of 4.5 m; blue ball, at groundwater depth of 11.0 m. *p < 0.05; **p < 0.01; ***p < 0.001.

The current‐year carbon pools influenced next‐year population dynamics (Figure 6). Soluble sugar, NSC, and total carbon pools from the current year were negatively correlated with next‐year individual biomass (*p* < 0.05, Figure 6a,g,j), but positively correlated with next‐year population biomass (*p* < 0.05, Figure 6i,k,l) and strongly positively correlated with next‐year population density (*p* < 0.01, Figure 6b,c,h). Among these, the total carbon pool exhibited the strongest explanatory power. In contrast, the current‐year starch pool showed no significant correlation with subsequent population density or biomass (Figure 6e,f), indicating that functional carbon components (soluble sugars and total carbon) preferentially support population recruitment rather than individual growth.

**FIGURE 7:**
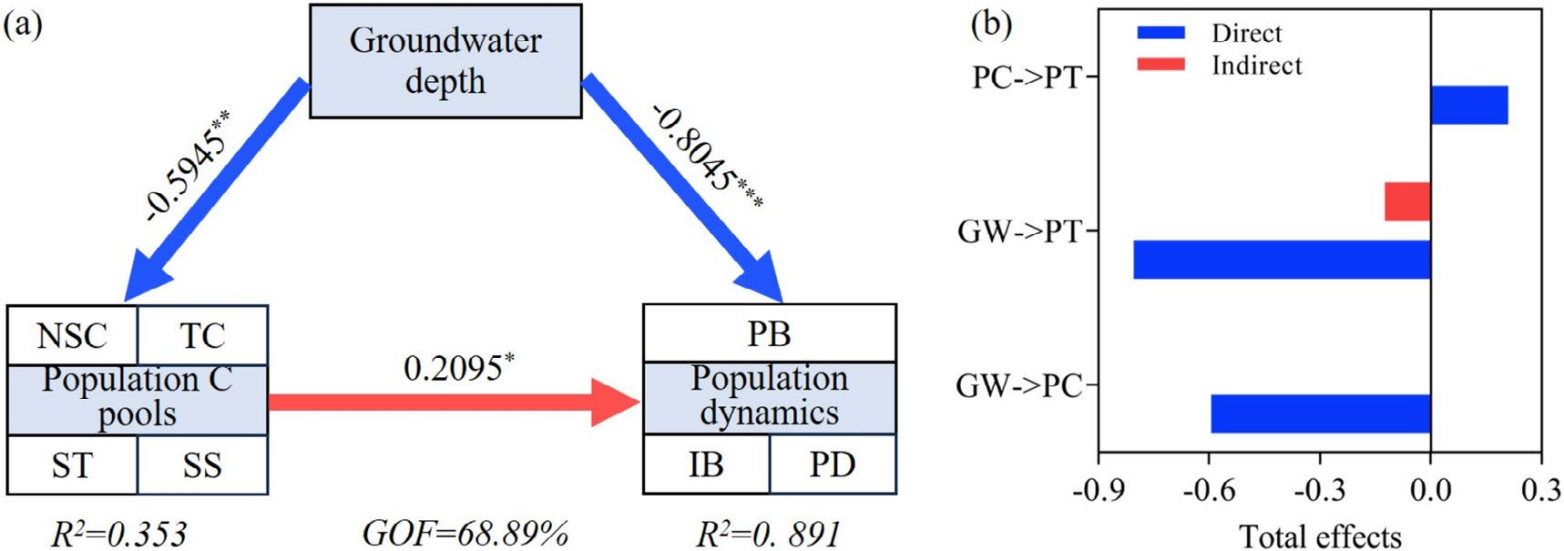
Partial least squares path modeling (PLS‐PM) for the effects of groundwater depth on the current population C pools and next‐year population density and biomass dynamics (a) and the total path effect of the PLS‐PM results (b). GOF, Goodness of fit; GW, groundwater depth. IB, individual biomass; PB, population biomass; PC, population C pools; PD, population density; PT, population dynamic traits; SS, soluble sugars; ST, starch; TC, total carbon. The red and blue lines represent negative and positive effects, respectively. *p < 0.05; **p < 0.01; ***p < 0.001.

PLS‐PM further quantified these relationships (GoF = 68.89%), revealing that groundwater depth suppressed population recruitment through two distinct pathways (Figure 7a). Groundwater depth directly negatively affected next‐year population density and biomass (path coefficient = −0.80, explaining 89.1% of the variance), and indirectly constrained them by reducing current‐year carbon pools (path coefficient = −0.60, explaining 35.3% of the variance), resulting in an indirect negative effect (indirect effect = −0.13). Although the current‐year carbon pools had a compensatory positive effect on next‐year population dynamics (path coefficient = 0.21, *p* < 0.05), they were insufficient to offset the overall negative impact of groundwater decline.

## 4 Discussion

### 4.1 Population Growth Responses to Groundwater Depth

Our findings underscore the pivotal role of groundwater depth in shaping the population dynamics and biomass of *A. sparsifolia*. Specifically, declining groundwater levels resulted in reduced population density but increased individual biomass, consistent with the classic “density–growth trade‐off” observed in desert perennials (e.g., *Haloxylon* spp., Xu et al. 2024; *Phragmites communis* , Zhao, Liu, et al. 2003). This trade‐off likely reflects adaptive resource allocation strategies, whereby density reduction alleviates intra‐specific competition and promotes individual‐level investment in root extension (Xu et al. 2017) and canopy efficiency (Xu et al. 2023), thus enhancing water acquisition (Mu et al. 2024). Two mechanisms may drive this population adjustment: (1) groundwater depletion reduced soil moisture (Figure S2; Malik et al. 2021), intensifying density‐dependent mortality through self‐thinning (Zhu et al. 2024); (2) water stress constrained clonal propagation (Zhang et al. 2013) and seedling recruitment (Liu et al. 2011), thereby limiting population renewal and shifting survival strategies from expansion to persistence (Si et al. 2011). The observed negative correlation between density and individual biomass (*r* = −0.64) further suggests a resource‐mediated trade‐off balancing individual performance with population persistence.

Furthermore, total population biomass declined significantly with increasing groundwater depth, driven primarily by reductions in population density (*r* = 0.88). This indicates a cascading, density‐dependent pathway through which hydrological constraints scale up to population‐level productivity. Two complementary mechanisms were proposed: (1) shallow groundwater enhanced water availability for mother ramets via deep roots (Thomas et al. 2000; Arndt et al. 2004), facilitating clonal support for daughter ramets (Luo et al. 2015); (2) reduced density under deep groundwater restricted canopy closure, lowering stand‐level carbon assimilation (Eamus et al. 2015; Lv et al. 2013). Despite compensatory biomass increases in surviving individuals, the overall decline in density appears to constrain population‐level carbon sequestration, with implications for vegetation stability in arid regions. Our results emphasize the need to incorporate groundwater thresholds into regional ecohydrological management to safeguard ecosystem function under increasing aridity.

### 4.2 NSC Dynamics and Their Ecophysiological Significance

NSCs serve as key metabolic reserves that buffer plants against environmental variability (McDowell et al. 2008). In *A. sparsifolia*, NSC contents in current‐year branches were significantly modulated by groundwater depth, seasonality, and interannual climate variability, suggesting a high degree of physiological plasticity. The nonlinear response of NSCs across groundwater gradients further indicates a finely tuned carbon allocation strategy under shifting hydrological conditions.

At intermediate groundwater depths (~4.5 m), both soluble sugar and starch contents declined, while total carbon content increased slightly, possibly reflecting translocation of photo assimilates to belowground organs to enhance water uptake (Martínez‐Vilalta et al. 2016). In contrast, at greater depths (11.0 m), soluble sugar and starch levels increased sharply (> 60%), while total carbon remained stable, indicating that NSC accumulation may facilitate osmotic regulation and drought resilience. Notably, individuals at 11.0 m had 172.9% greater biomass than those at 2.5 m, suggesting that low‐density conditions promote individual size increase to maintain photosynthetic efficiency and minimize resource competition.

Seasonal variation in NSC composition was also marked. Soluble sugar and total NSC contents peaked in winter, likely enhancing cold tolerance via osmotic adjustments (Morin et al. 2007). Starch peaked before budburst and declined sharply during early growth, indicating remobilization of stored reserves to support new tissues (Bréda et al. 2006; Furze et al. 2019). The low SS/ST ratio in April (0.92%) confirms active starch‐to‐sugar conversion during high metabolic demand.

Correlation analysis revealed that the dynamics of NSC contents were largely decoupled from population density and biomass. Soluble sugar and NSC contents, as well as the NSC/TC (%), were consistently positively correlated (*r* ^2^ > 0.65) across seasons, indicating that soluble sugars predominantly regulate NSC fluctuations. Although NSCs were positively correlated with branch water content (*r* = 0.75), they showed no significant relationship with individual or population biomass or density, suggesting that NSC accumulation primarily serves to maintain cellular water homeostasis (Dietze et al. 2014) rather than directly promoting growth. This finding highlights a fundamental disconnect between carbon storage and morphological expression in this species, emphasizing the complexity of interpreting NSC metrics as indicators of growth under stress conditions.

### 4.3 Carbon Pool Allocation and Population‐Level Consequences

Our results revealed contrasting patterns of carbon allocation at individual versus population levels in response to groundwater changes. At the individual scale, groundwater decline led to significant increases in NSC and total carbon pools, with soluble sugars comprising 62.3% of the NSC fraction. This likely reflects an adaptive shift toward osmotic regulation and hydraulic safety under drought stress (Dietze et al. 2014; Song et al. 2020). In contrast, population‐level carbon pools, biomass, and density declined sharply beyond 4.5 m groundwater depth, suggesting a critical ecological threshold where *A. sparsifolia* transitions from “growth and reproduction” to “maintenance” mode (Eamus et al. 2015). This finding aligns with prior studies identifying 2–4 m as the optimal groundwater depth for this species (Liu et al. 2006).

Interannual variation in NSC pools—driven largely by temperature—further illustrates the dual influence of groundwater and climate on carbon metabolism. Soluble sugars increased in warmer years, while starch accumulated under cooler conditions, consistent with thermal regulation of amylase activity (Du et al. 2020; Klopotek and Kläring 2014). These bidirectional shifts underscore the role of temperature in modulating storage form and function, with implications for seasonal carbon availability.

NSC pools, especially soluble sugars and total carbon, showed significant positive correlations with next‐year population density and biomass, affirming the importance of carbon reserves in recruitment and growth (von Arx et al. 2017; Palacio et al. 2018). Interestingly, starch pools contributed little to population expansion, suggesting that soluble sugars act as the primary mobile carbon currency during early regeneration. Ecologically, the population carbon pool was more tightly correlated with density than biomass, and negatively correlated with individual biomass. This indicates a shift toward “quantity over size” as a drought adaptation, whereby carbon resources are preferentially allocated to sustain recruitment rather than individual growth. Such a strategy may enhance population resilience in unpredictable, resource‐pulsed environments.

Our PLS‐PM (GoF = 68.89%) supports a strong coupling between water availability, carbon metabolism, and demographic responses. Groundwater depth exerted both direct and indirect (via carbon pool suppression) negative effects on population dynamics. Notably, a “carbon–water synergy threshold” was observed at ~4.5 m, beyond which water limitation outweighed carbon storage benefits. These results highlight that the long‐term viability of groundwater‐dependent species like *A. sparsifolia* hinges on maintaining a functional balance between resource storage and acquisition. Integrating such mechanistic understanding into conservation planning is critical for managing arid ecosystems under accelerating climate change.

## 5 Conclusions

Our study reveals that groundwater depth is a key factor in regulating the population dynamics, biomass, and carbon storage of *A. sparsifolia* in hyper‐arid environments. Groundwater decline was found to be the dominant driver of vegetation dynamics, surpassing climate variability. Soluble sugars play a crucial role in short‐term water stress tolerance, but long‐term survival depends on the total carbon pool, which is constrained by groundwater availability. A threshold of 4.5 m groundwater depth marks the shift from growth to maintenance‐driven survival. Additionally, *A. sparsifolia* employs a hierarchical adaptive strategy that balances individual carbon accumulation with communal water‐sharing. As groundwater declines, the ecosystem's carbon sink capacity diminishes, increasing vulnerability to degradation. Our findings highlight the need for integrated ecohydrological management to ensure ecosystem stability in water‐limited environments and support sustainable vegetation restoration and carbon management in hyper‐arid regions.

## Author Contributions

**Caibian Huang:** funding acquisition (lead), investigation (lead), project administration (lead), writing – original draft (lead). **Zhiqiang Ma:** formal analysis (lead), investigation (equal). **Bo Zhang:** data curation (lead), software (equal). **Fanjiang Zeng:** resources (lead), supervision (lead). **Shaomin Zhang:** funding acquisition (equal), software (equal), writing – review and editing (lead).

## Conflicts of Interest

The authors declare no conflicts of interest.

## Supporting information


**Data S1:** ece372395‐sup‐0001‐DataS1.csv.

**Data S2:** ece372395‐sup‐0002‐DataS2.csv.

**Figures S1–S4:** ece372395‐sup‐0003‐FiguresS1‐S4.docx.

## Data Availability

We have shared our data in the Supporting Information (Data S1 and S2).
