# Supplementary material for: Groundwater Depth Drives Carbon Pools and Population Dynamics in Deep‐Rooted Desert Plants of a Hyper‐Arid Ecosystem
Source: Ecol Evol. 2025 Oct 30;15(11):e72395. doi: 10.1002/ece3.72395 (PMC12572830; doi:10.1002/ece3.72395)
Supplement: Supplementary file 3 — Figures S1–S4: ece372395‐sup‐0003‐FiguresS1‐S4.docx. [file ECE3-15-e72395-s003.docx]

**Groundwater Depth Drives Carbon Pools and Population Dynamics in Deep-Rooted Desert Plants of a Hyper-Arid Ecosystem**


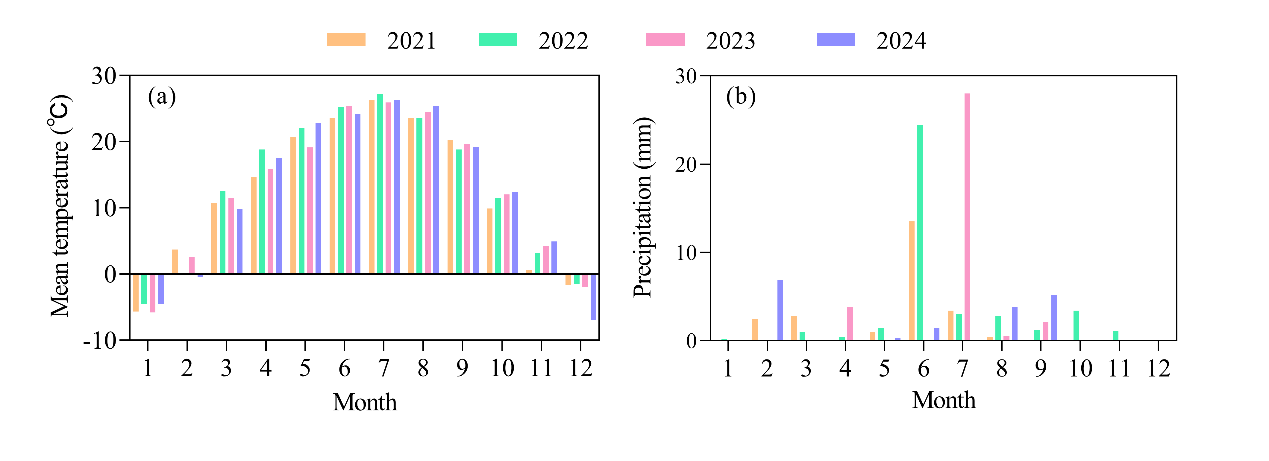


Fig.S1. Monthly precipitation (a) and mean temperature (b) at the study site in 2021, 2022, 2023, and 2024.


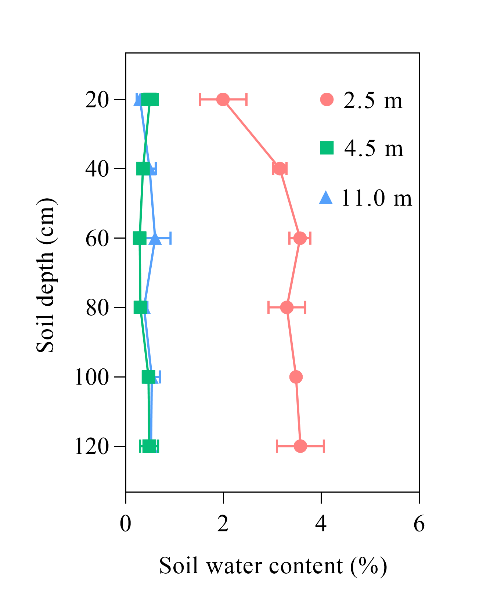


Fig.S2. Soil water content changes in profiles at different groundwater depths, based on the 2021–2023 average.


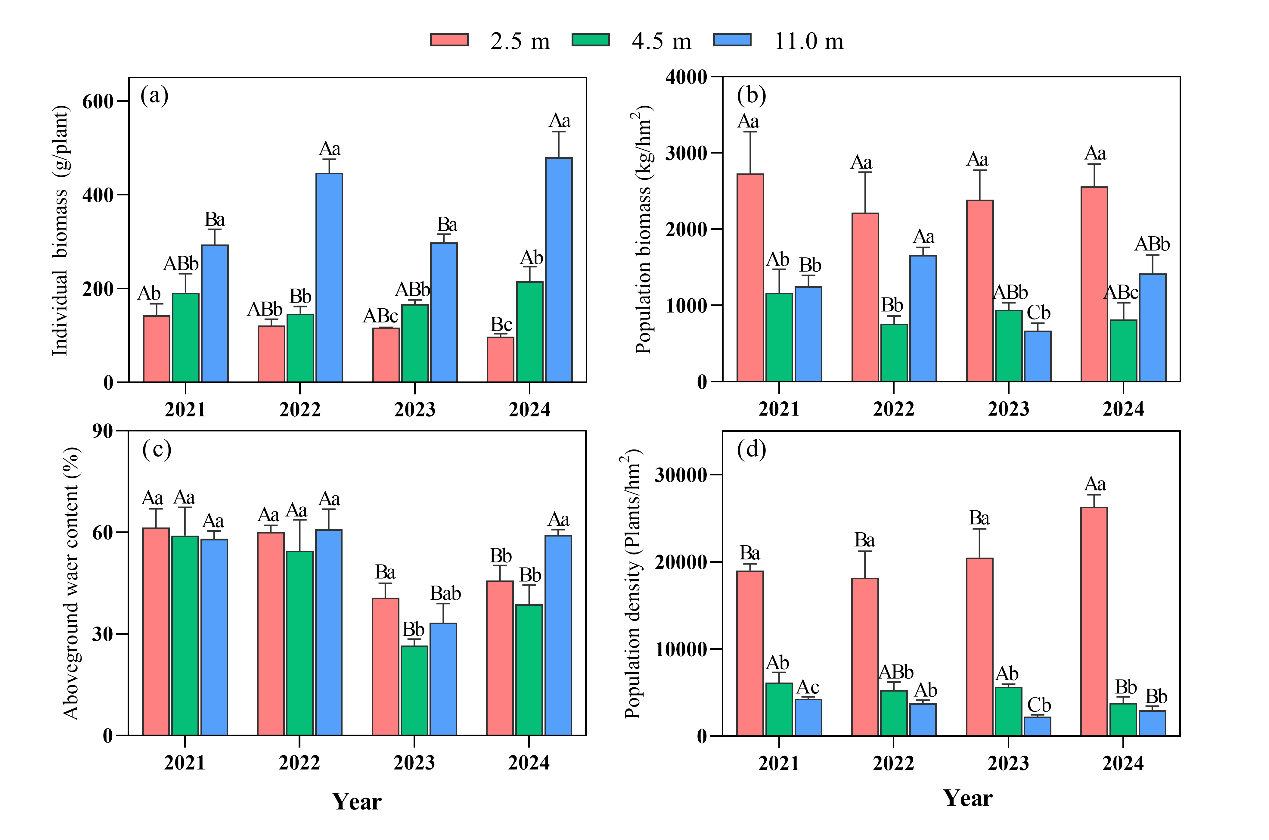


Fig.S3. Variations in individual and population biomass, population density, and aboveground water content in 2021, 2022, 2023, and 2024 at different groundwater depths and years (mean ± SE). Different small letters represent significant differences among groundwater depth at *P* < 0.05. Different capital letters represent significant differences among years at *P* < 0.05.


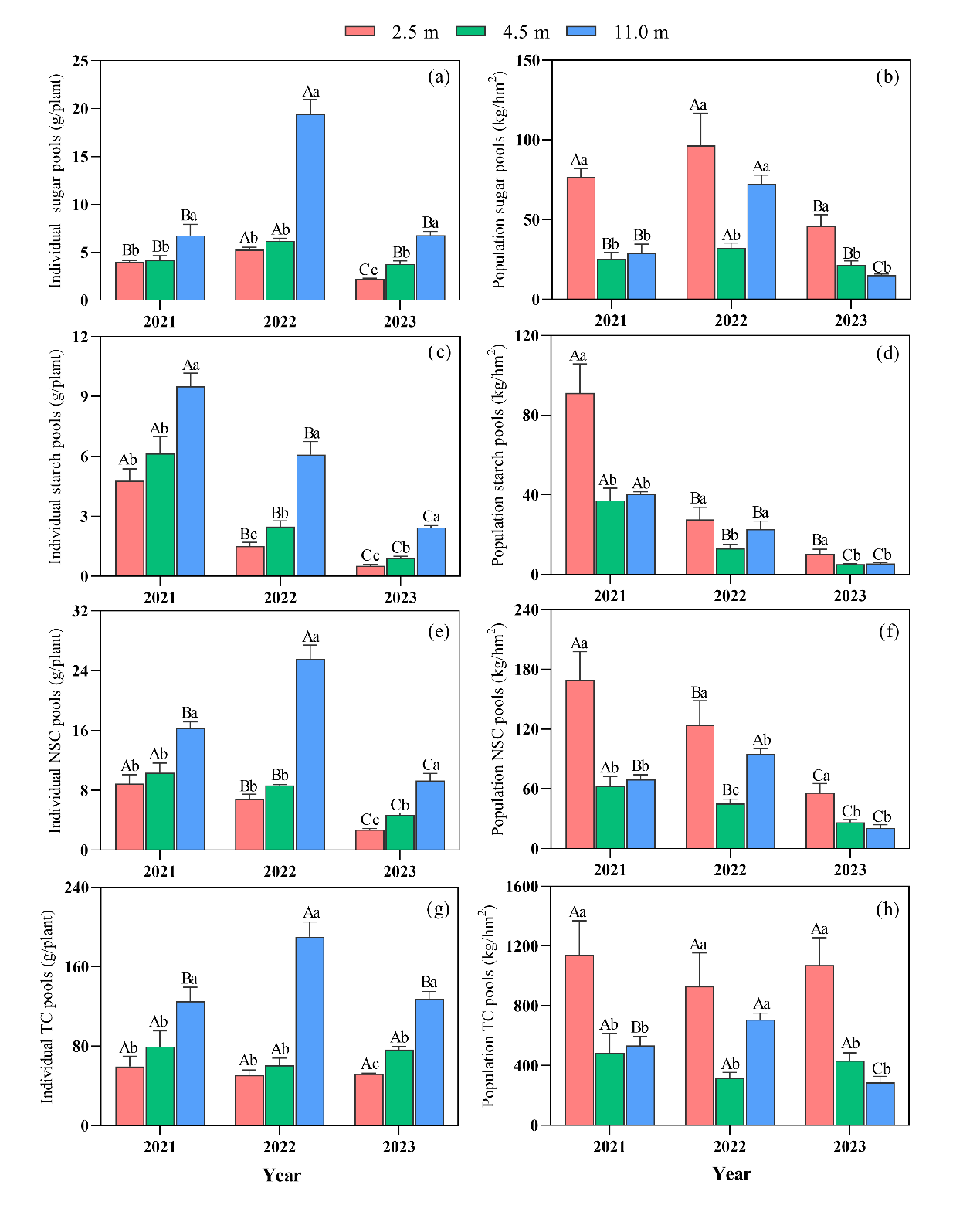


Fig.S4. Variations in the individual and population C pools in 2021, 2022 and 2023 at different groundwater depths and years (mean ± SE). Different small letters represent significant differences among groundwater depths at *P* < 0.05. Different capital letters represent significant differences among years at *P* < 0.05.
